# Dual-Channel Fluorescent/Colorimetric-Based OPD-Pd/Pt NFs Sensor for High-Sensitivity Detection of Silver Ions

**DOI:** 10.3390/foods12234260

**Published:** 2023-11-24

**Authors:** Yuan Fang, Shusen Ding, Weiran Li, Jingjing Zhang, Hui Sun, Xiaodong Lin

**Affiliations:** 1 State Key Laboratory of Food Nutrition and Safety, Tianjin Key Laboratory of Food Quality and Health, College of Food Science and Engineering, Tianjin University of Science and Technology, Tianjin 300457, China; fangyuan@tust.edu.cn (Y.F.); dingshusen66@mail.tust.edu.cn (S.D.); jingjingzhang@mail.tust.edu.cn (J.Z.); 23842003@mail.tust.edu.cn (H.S.); 2 Zhuhai UM Science & Technology Research Institute, Zhuhai 519000, China

**Keywords:** silver ions, Pd/Pt nanoflowers, fluorescent sensor, colorimetric sensor

## Abstract

Silver ions (Ag^+^) exist widely in various areas of human life, and the food contamination caused by them poses a serious threat to human health. Among the numerous methods used for the detection of Ag^+^, fluorescence and colorimetric analysis have attracted much attention due to their inherent advantages, such as high sensitivity, simple operation, short time, low cost and visualized detection. In this work, Pd/Pt nanoflowers (NFs) specifically responsive to Ag^+^ were synthesized in a simple way to oxidize o-phenylenediamine (OPD) into 2,3-diaminophenazine (DAP). The interaction of Ag^+^ with the surface of Pd/Pt NFs inhibits the catalytic activity of Pd/Pt NFs towards the substrate OPD. A novel dual-channel nanosensor was constructed for the detection of Ag^+^, using the fluorescence intensity and UV-vis absorption intensity of DAP as output signals. This dual-mode analysis combines their respective advantages to significantly improve the sensitivity and accuracy of Ag^+^ detection. The results showed that the limit of detection was 5.8 nM for the fluorescence channel and 46.9 nM for the colorimetric channel, respectively. Moreover, the developed platform has been successfully used for the detection of Ag^+^ in real samples with satisfactory recoveries, which is promising for the application in the point-of-care testing of Ag^+^ in the field of food safety.

## 1. Introduction

With the rapid development of global industrialization, the content of various heavy metals in certain environments has exceeded the normal range, directly or indirectly endangering human health worldwide [1,2]. Many harmful organic compounds can be reduced or eliminated via the physical, chemical or biological purification of nature itself. In contrast, heavy metals have enrichment properties and are difficult to degrade in the environment [3,4]. As one of the common heavy metals, silver and its compounds are widely used in industries such as pharmaceuticals, electrical and photography. However, the large amount of silver released into the surrounding environment from industrial waste each year is becoming a growing problem. Public health faces enormous challenges due to the potential contamination of food and water by silver ions (Ag^+^) present in daily life [5,6,7]. Ag^+^ pollution in food comes partly from the enrichment of Ag^+^ in crops and partly from the pollution that occurs during food production and transportation [8,9]. Ag^+^ is also often used to disinfect drinking water due to its strong antibacterial activity at low concentrations. However, residual Ag^+^ in water can accumulate in the human body through direct consumption and the food chain [10,11,12]. Excessive levels of Ag^+^ in the body may harm human health and even cause adverse symptoms, such as skin infections, diarrhea and nerve damage [13,14]. As a consequence, the detection of Ag^+^ in easily contaminated food and water is of great practical significance for evaluating food quality, protecting human health and maintaining sustainable socio-economic development [15,16].

Currently, the commonly used detection methods for Ag^+^ include inductively coupled plasma mass spectrometry (ICPMS), voltammetry, biochemical sensors and enzyme inhibitor methods [17,18,19]. Although these methods can accurately detect the content of Ag^+^, they require expensive instruments, complex operations and lengthy detection processes [20,21]. In stark contrast, fluorescent and colorimetric analyses are very suitable for the rapid detection of Ag^+^ due to their inherent advantages of high sensitivity, simple operation, short time, low cost and visualized detection [22]. Detecting Ag^+^ through two different modes can effectively avoid false-positive and false-negative results, expanding the application range. It is worth noting that some nanoparticles with enzyme-like activity can catalyze substrates to simultaneously bring about fluorescence and color changes, which is promising for using this appropriate mechanism to accurately detect Ag^+^ [23]. For example, the nanoparticles formed by Pd and Pt in platinum group metals have similar physical and chemical properties, and they both have excellent peroxide-like properties. Compared to single Pd or Pt nanoparticles, Pd/Pt bimetallic nanoparticles exhibit synergistic catalytic effects and, therefore, have higher catalytic activity [24]. Moreover, Pd/Pt nanoparticles have the beneficial characteristics of simple synthesis, low cost, good stability and convenient storage, as they can bind specifically to Ag^+^ in solution [25].

**Scheme 1:**
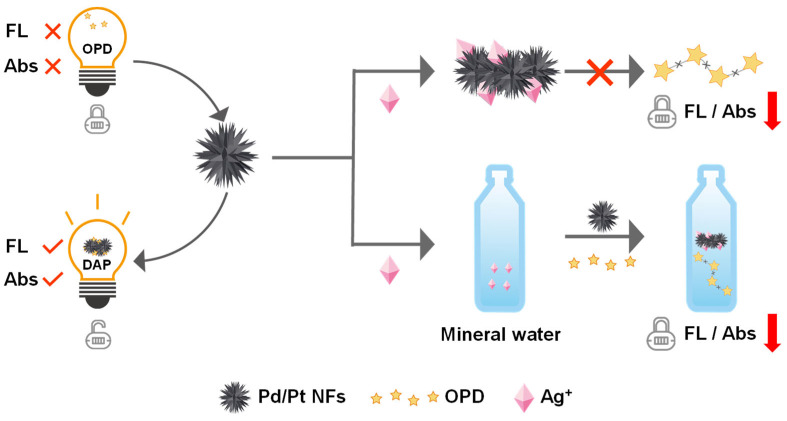
The principles of the fluorescent and colorimetric dual-channel nanosensor for the detection of Ag^+^**.** OPD can catalyze the generation of DAP through Pd/Pt NFs that possess peroxidase activity. In the presence of Ag^+^, the catalytic activity of Pd/Pt NFs is specifically inhibited. The concentration of Ag^+^ in the sample is determined via the monitoring of the fluorescence intensity and UV-vis absorption from DAP.

In this work, a novel dual-channel nanosensor using fluorescence intensity and ultraviolet (UV) absorption as signals was constructed for the highly sensitive and specific detection of Ag^+^ (Scheme 1). At first, Pd/Pt nanoflowers (NFs) with excellent peroxidase-like activity were synthesized via the simple one-pot method. Pd/Pt NFs can effectively oxidize o-phenylenediamine (OPD) in colorless and transparent solution to 2,3-diaminophenazine (DAP). DAP not only appears light yellow in solution but also emits orange fluorescence under 414 nm laser excitation. When Ag^+^ is present in solution, Ag^+^ can specifically interact with the surface of Pd/Pt NFs, thereby weakening the catalytic ability of Pd/Pt NFs to substrate OPD. The reduction in DAP generation leads to a simultaneous decrease in fluorescence intensity and UV absorption, which are used as output signals to construct a dual-channel sensor for the highly sensitive detection of Ag^+^. The results showed that the limit of detection was 5.8 nM for the fluorescence channel and 46.9 nM for the colorimetric channel, respectively, which is much less than the maximum concentration (0.05 mg L^−1^, 464 nM) suggested by the United States Environmental Protection Agency (EPA), as well as the maximum concentration (0.1 mg L^−1^, 927 nM) suggested for drinking water by the World Health Organization (WHO) [3]. Furthermore, the accurate detection of Ag^+^ in real samples of commercial mineral water is subsequently achieved. The developed detection method has important practical application value in terms of preventing food safety issues caused by excessive Ag^+^ content in food.

## 2. Materials and Methods

### 2.1. Reagents and Instruments

O-phenylenediamine (OPD), H_2_PtCl_6_, K_2_PdCl_4_ and H_2_PdCl_4_ were purchased from Shanghai Aladdin Biochemical Science and Technology Co., Ltd. (Shanghai, China). HCl, NaH_2_PO_4_, Na_2_HPO_4_, KH_2_PO_4_, NaOH, CoCl_2_, ascorbic acid (AA), MES, Tris and HEPES were purchased from Shanghai Sinopharm Chemical Reagent Co., Ltd. (Shanghai, China). NaCl and CaCl_2_ were purchased from Sinopharm Group Supply Chain Co., Ltd. (Tianjin, China). Pluronic F127 and Zn(COOH)_2_ were from Shanghai Yuanye Biotechnology Co., Ltd. (Shanghai, China). AgNO_3_, KCl, Mn(NO_3_)_2_, FeCl_2_·4H_2_O and CrCl_3_ were obtained from Tianjin Tiangan Chemical Technology Co., Ltd. (Tianjin, China). All the reagents and chemicals were of analytical reagent grade.

Ultrapure water was prepared using the Millipore ultrapure water system (Merck, Shanghai, China). Transmission electron microscopy (TEM) measured the morphology and elements of the nanomaterials (FEI, Lausanne, PA, USA). The fluorescence spectrophotometer detected the fluorescence intensity in the range from 500 nm to 670 nm (Thermo Scientific, Saint Louis, MO, USA). The UV-Vis spectrophotometer detected absorbance in the range from 330 nm to 600 nm (Shimadzu, Shanghai, China).

### 2.2. Preparation of OPD Solution and Pd/Pt NFs

Next, 0.00432 g of OPD was dissolved in 1 mL of ultrapure water to form an OPD solution (40 mM). After thorough mixing, the OPD solution was stored at 4 °C for further use.

Pd/Pt NFs were prepared using a modified method previously reported in [26]. H_2_PdCl_4_ (1 mL, 20 mM), H_2_PtCl_6_ (1 mL, 20 mM), K_2_PdCl_4_ (1 mL, 20 mM), HCl (120 μL, 20 mM) and 60 mg of Pluronic F127 were added into a glass bottle under vigorous stirring. After the above reagents were completely mixed, AA (3 mL, 0.1 M) was subsequently added into the solution. The mixed solution was sonicated for 4 h and centrifuged at 14,000 rpm for 20 min to obtain the precipitate. Finally, the resulting Pd/Pt NFs were washed three times with ethanol and water, respectively, and then stored at 4 °C for further use.

### 2.3. Fluorescence and Colorimetric Dual-Channel Detection of Ag^+^

Pd/Pt NFs (10 μL, 1 μg/mL) and different concentrations of AgNO_3_ (10 μL, 0–100 μM) were mixed well and incubated at 25 °C for 40 min. After the complete interaction between Ag^+^ and Pd/Pt NFs, OPD (10 μL, 20 mM) and ultrapure water (170 μL) were added into the above solution. OPD was catalyzed to generate DAP after incubation at 25 °C for 40 min. The mixtures were excited using an excitation wavelength of 414 nm, and their fluorescence intensity profiles were detected from 500 nm to 670 nm. Their UV absorption profiles were detected from 330 nm to 600 nm. The detection range of Ag^+^ concentration was determined according to the measurement results of fluorescence intensity at 563 nm and UV absorption at 418 nm, followed by calculating the lowest detection limit of Ag^+^. At the same time, the color changes in the mixed solution under UV and natural light were all recorded by taking photos with a smartphone.

To evaluate the impact of buffers on the system, OPD (10 μL, 20 mM), Pd/Pt NFs (10 μL, 1 μg/mL) and different buffers (including ultrapure water, PB, MES, PBS and HEPES, 180 μL) were each mixed. After incubating the mixtures at 25 °C for 40 min, the fluorescence signal and UV absorption of the systems were then recorded. To further evaluate the optimal reaction time of OPD and Pd/Pt NFs, the mixture was firstly prepared by mixing OPD (10 μL, 20 mM), Pd/Pt NFs (10 μL, 1 μg/mL) and ultrapure water (180 μL). The fluorescence intensity and UV absorption of the systems were then recorded at 25 °C after incubation for different times (from 0 to 50 min). Similarly, to determine the optimal interaction time of Ag^+^ with Pd/Pt NFs, Ag^+^ (10 μL, 100 μM) and Pd/Pt NFs (10 μL, 1 μg/mL) were mixed and incubated 25 °C for different times (from 0 to 60 min). After incubation, OPD (10 μL, 20 mM) and ultrapure water (170 μL) were added to the above solution and incubated for 40 min. Both the fluorescence intensity and UV adsorption of the systems were subsequently measured.

### 2.4. Selectivity of Ag^+^ Detection

To exclude the impact of other metal ions on the accuracy of this detection method, Ag^+^ in the systems was replaced with Mn^2+^, K^+^, Zn^2+^, Co^2+^, Na^+^, Ca^2+^, Cr^3+^ and Fe^2+^ (100 μM), respectively. Under the same experimental conditions (25 °C, 40 min), Ag^+^ specificity was evaluated based on the corresponding fluorescence intensity and UV absorption results.

### 2.5. Ag^+^ Detection in Real Samples

To investigate the feasibility and accuracy of this method in real samples, three types of mineral water from different manufacturers were purchased from the nearby supermarket and validated. To simulate Ag^+^ contamination in food, AgNO_3_ solutions of different concentrations (2 and 4 μM) were prepared using mineral water. Three parallel controls were set up in each experimental group. The changes in fluorescence intensity and UV absorption in different mineral water systems were measured. The concentrations, recovery rates and relative standard deviations of Ag^+^ in real samples were calculated based on linear equations.

## 3. Results

### 3.1. Characterization of Pd/Pt NFs

**Figure 1:**
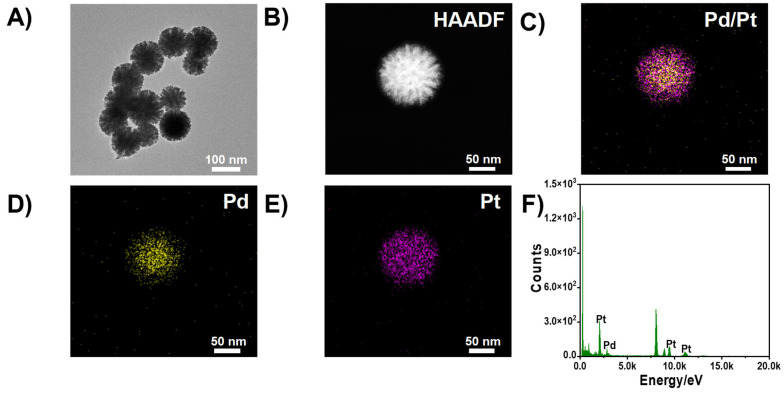
(**A**) TEM images of Pd/Pt NFs. HAADF (**B**) of Pd/Pt NFs and corresponding element mapping (**C**–**E**) analysis of Pd/Pt NFs. (**F**) The EDS of Pd/Pt NFs.

The Pd/Pt NFs were synthesized in a simple way and then characterized via transmission electron microscopy (TEM). As shown in Figure 1A, the TEM image shows that the as-synthesized Pd/Pt NFs are spherical and have nanoflower-like morphology with uniform distribution. The average particle size of Pd/Pt NFs is about 96 nm using statistical analysis. The high angle annular dark field (HAADF) is a dark field diagram, which can obtain the finer morphology of Pd/Pt NFs. Figure 1B further shows that the structures of Pd/Pt NFs are fluffy and, therefore, have larger surfaces. The compositions of Pd/Pt NFs are analyzed via the EDS mapping technique (Figure 1C–E). Both Pd and Pt Elements are well dispersed in the particle. In addition, the energy dispersive spectrometer (EDS) clearly shows peaks for Pd and Pt (Figure 1F). The above data demonstrate the successful preparation of Pd/Pt NFs.

### 3.2. Verification of Ag^+^ Dual-Channel Sensing Mechanism

**Figure 2:**
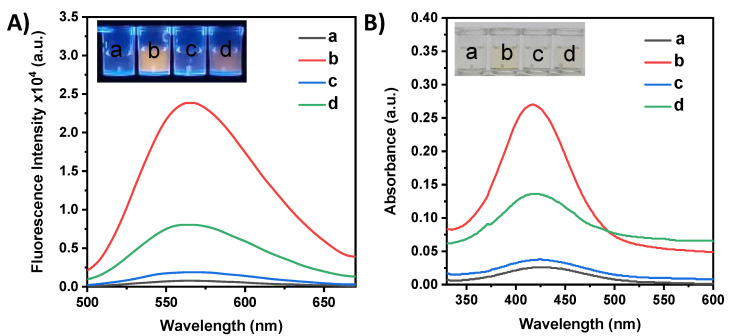
(**A**) Fluorescence spectra and insets under UV light and (**B**) UV-vis absorption spectra and insets under daylight based on the mechanism validation of Ag^+^ detection for OPD-Pd/Pt NFs (a: OPD, b: OPD-Pd/Pt NFs, c: OPD-Ag^+^, d: OPD-Pd/Pt NFs-Ag^+^).

The Pd/Pt NFs possess excellent peroxidase-like activity, meaning that OPD can be efficiently oxidized by them to form DAP. According to the previous experimental results of our research group, the distribution of Ag and Pd elements showed good co-locality. The coupling of Ag and Pd elements can inactivate the catalytic activity of Pd/Pt NFs [27]. As shown in Figure 2A,B, the OPD solution without Pd/Pt NFs or Ag^+^ is colorless and transparent, producing little fluorescence. In contrast, the fluorescence of the mixed solution containing OPD and Pd/Pt NFs is significantly enhanced, accompanied with a characteristic emission peak at 563 nm. The color of the mixed solution is found to be orange under UV light, further implying that Pd/Pt NFs can successfully catalyze OPD into DAP (Figure 2A, line b and inset b). When Ag^+^ is added into the above solution, the fluorescence intensity clearly decreases, and the orange solution becomes lighter (Figure 2A, line d and inset d). These results indicate that Ag^+^ can effectively inhibit the catalytic activity of Pd/Pt NFs and reduce the rate of OPD oxidation.

Similarly, the mixed solution of OPD and Pd/Pt NFs exhibits enhanced UV-vis absorption via a characteristic peak at 418 nm, and the color of the solution can be observed to be light yellow in daylight (Figure 2B, line b and inset b). The addition of Ag^+^ results in a significant decrease in the UV-vis absorption of the mixed solution, and the color of the mixed solution becomes lighter (Figure 2B, line d and inset d). Moreover, in the absence of Pd/Pt NFs, the OPD solution co-incubated with Ag^+^ undergoes no obvious change in fluorescence intensity and UV-vis absorption (Figure 2A,B, line c and inset c), showing that OPD and Ag^+^ hardly directly react to generate DAP. Thus, the above data clearly prove that fluorescence and UV-vis absorption simultaneously serve as output signals to detect Ag^+^ based on the OPD-Pd/Pt NFs system. Moreover, the mutual consistency of the two modes provides for the reliability of Ag^+^ detection.

### 3.3. Optimization of Ag^+^ Detection Conditions

**Figure 3:**
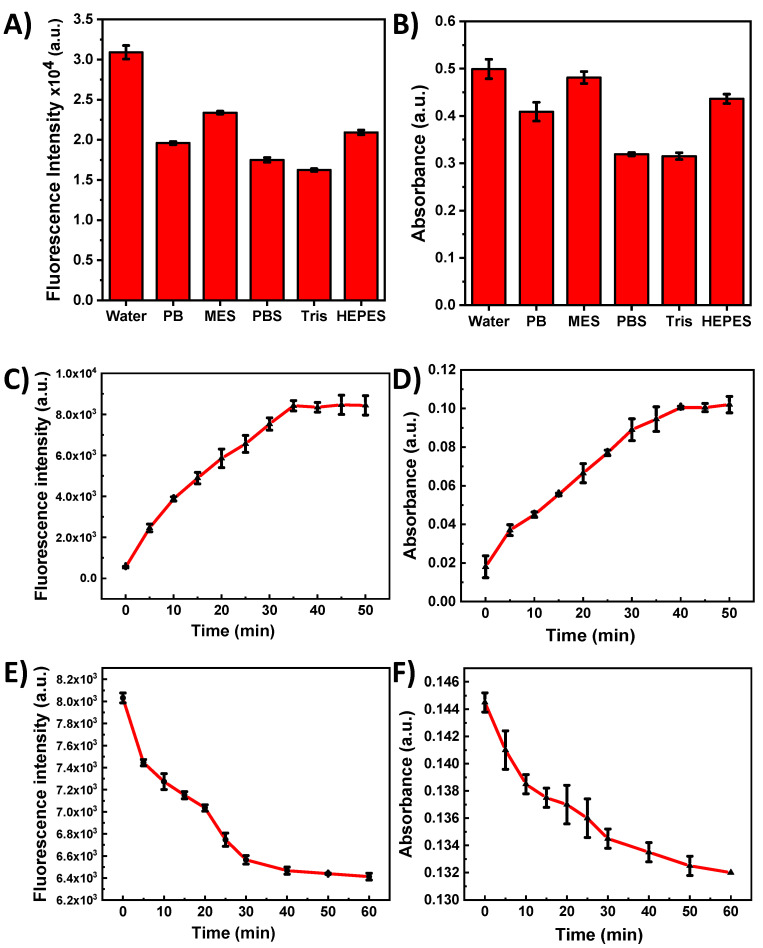
(**A**) Fluorescence intensity and (**B**) UV-vis absorption of OPD-Pd/Pt NFs detection system under different buffers. (**C**) Optimization of reaction time between OPD and Pd/Pt NFs based on fluorescent analysis. (**D**) Optimization of reaction time between OPD and Pd/Pt NFs based on colorimetric analysis. (**E**) Optimization of incubation time between Pd/Pt NFs and Ag^+^ based on fluorescent analysis. (**F**) Optimization of incubation time between Pd/Pt NFs and Ag^+^ based on colorimetric analysis.

In order to obtain the better analytical performance of the as-constructed dual-channel sensor, the following the critical factors were optimized separately, including the reaction buffer and incubation time for dual-mode platform. Firstly, the effects of different buffers on the sensitivity of Ag^+^ detection were investigated by monitoring the fluorescence intensity and UV-vis absorption of DAP. As shown in Figure 3A,B, compared to other buffers (including PB, MES, PBS, Tris and HEPES), DAP in water has the strongest fluorescence signal and UV absorption. The fluorescence intensity and UV absorption of other buffers decreases to different degrees, possibly due to various ions in these buffers interfering with the catalytic activity of Pd/Pt NFs. Hence, water was chosen as the optimal buffer for Ag^+^ sensing.

The incubation time of OPD and Pd/Pt NFs is another key factor for the accurate detection of Ag^+^. To ensure that OPD was fully oxidized, their incubation time was evaluated via the monitoring of the fluorescence intensity and UV absorption signals of the mixed solution. As shown in Figure 3C, the fluorescence of OPD solution itself is negligible. With the prolongation of the incubation time of OPD and Pd/Pt NFs, the fluorescence intensity of the mixed solution gradually increases. When the reaction time lasts 40 min, the fluorescence of the solution containing OPD and Pd/Pt NFs no longer increases, indicating that OPD has been completely oxidized to DAP. Meanwhile, the UV absorption of the mixed solution also reaches its maximum after incubating OPD with Pd/Pt NFs for 40 min (Figure 3D). Therefore, 40 min was set as the optimal incubating time for OPD and Pd/Pt NFs.

When Ag^+^ exist in the solution, they can interact with Pd/Pt NFs to inhibit the oxidation of OPD. In this regard, determining the incubation time between Ag^+^ and Pd/Pt NFs is also crucial for optimizing Ag^+^ sensing. As depicted in Figure 3E, Pd/Pt NFs rapidly oxidize OPD to produce the strongest fluorescence in the absence of Ag^+^. As Ag^+^ gradually bind to the surface of Pd/Pt NFs, the fluorescence intensity of the mixed solution shows a decreasing trend. Similarly, the UV absorption of the mixed solution continuously reduces with the co-incubation of Ag^+^ and Pd/Pt NFs (Figure 3F). Combining the results of fluorescence and UV, 50 min is set as the most appropriate incubation time for Ag^+^ and Pd/Pt NFs. In summary, ultrapure water was used as a buffer, the incubation time of samples containing Ag^+^ with Pd/Pt NFs was 50 min and the reaction time for OPD oxidation was 40 min, which were selected as the optimal conditions for subsequent Ag^+^ detection.

### 3.4. Selectivity Evaluation

**Figure 4:**
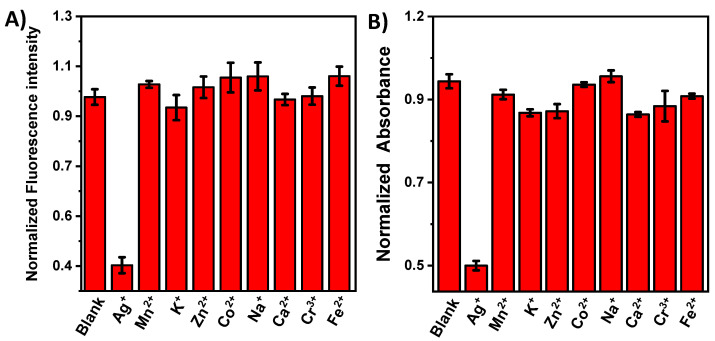
(**A**) Fluorescence intensity and (**B**) UV-vis absorption of a OPD-Pd/Pt NF detection system in the presence of different metal ions (including Ag^+^, Mn^2+^, K^+^, Zn^2+^, Co^2+^, Na^+^, Ca^2+^, Cr^3+^ and Fe^2+^). The concentration of metal ions was 100 μM.

In practical application, high selectivity for Ag^+^ sensing is necessary to avoid erroneous results. Selectivity evaluation involves determining the ability of the sensing system to specifically detect and distinguish the target metal ions from other metal ions that may be present in the sample. Ensuring the accurate and reliable detection of target metal ions is essential without interference from other ions. In order to evaluate the specificity of this detection method for Ag^+^, the effects of potential interfering substances (such as Mn^2+^, K^+^, Zn^2+^, Co^2+^, Na^+^, Ca^2+^, Cr^3+^ and Fe^2+^) on the fluorescence intensity and UV-vis absorption response of the solution were investigated. As shown in Figure 4A, the fluorescence intensity of the target Ag^+^ is the lowest compared to those of other substances, which indicates that only Ag^+^ can inhibit the catalytic performance of Pd/Pt NFs and, thus, realize the fluorescence-based strategy for target detection. On the other hand, the catalytic properties of the Pd/Pt NFs are suppressed and, consequently, the oxidation intensity of the OPD is reduced, again showing the lowest intensity on the UV-vis absorption (Figure 4B). Thus, there is good agreement between fluorescence and UV-vis, demonstrating the ability of our dual-mode method to specifically detect silver ions. Moreover, these two modes can verify each other, which lays the foundation for the specific detection of silver ions in real samples.

### 3.5. Ag^+^ Detection Using the Fluorescent and Colorimetric Dual-Mode Analysis

**Figure 5:**
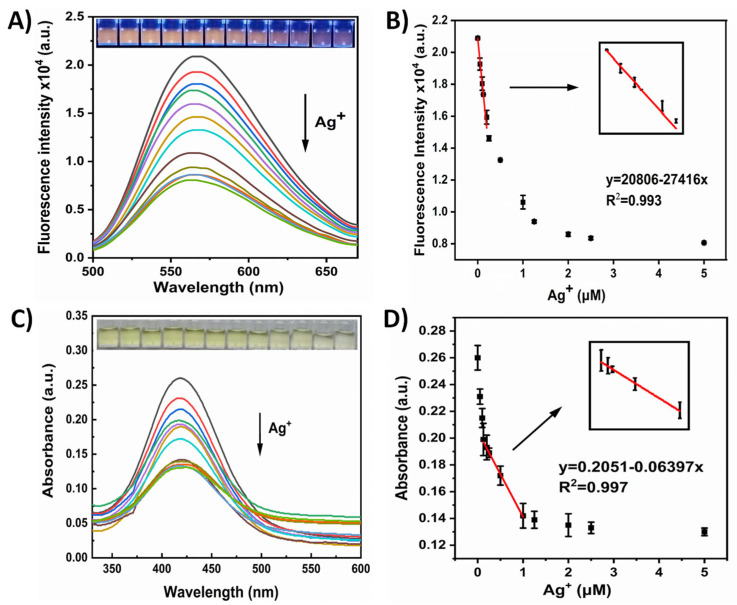
(**A**) Fluorescence spectra and insets under UV light and (**C**) UV-vis absorption spectra and insets under daylight in the sensing system in the presence of different concentrations of Ag^+^ (the concentrations from the black line to the green line are 0, 0.05, 0.1, 0.125, 0.2, 0.25, 0.5, 1, 1.25, 2, 2.5 and 5 μM, respectively). Standard curves based on the fluorescence intensity (**B**) and UV absorption (**D**) of the corresponding Ag^+^ concentrations.

Under the best conditions, Ag^+^ determination was performed using the mixed solution containing Pd/Pt NFs and OPD as the sensing system. As shown in Figure 5A, the fluorescence intensity of the initial system is relatively high in the absence of Ag^+^. After different concentrations of Ag^+^ are introduced, the fluorescence intensity of the sensing system gradually decreases with the increase in Ag^+^ concentrations. Notably, the linear relationship between the Ag^+^ concentrations ranging from 0 to 0.25 μM and the fluorescence intensity of the system at 563 nm can be acquired: y = 20,806 − 27,416x, R^2^ = 0.993 (Figure 5B). The LOD of the fluorescent channel is calculated to be 5.8 nM, showing that the developed fluorescence channel exhibits high sensitivity for the detection of Ag^+^. Interestingly, the quantitative detection of Ag^+^ can also be realized through the determination of UV-vis absorption spectra. Similarly, as shown in Figure 5C,D, the absorbance of the system at 418 nm gradually decreases with the increased Ag^+^ concentrations. The change is linear as the Ag^+^ concentrations varied from 0.125 to 1 µM (y = 0.2051 − 0.06397x, R^2^ = 0.997) with a LOD of 46.9 nM. As shown in Table 1, our proposed strategy for detecting Ag^+^ shows high sensitivity compared to other reported methods. Moreover, the signal changes induced via Ag^+^ introduction can also be directly observed with the naked eye. The yellow–red fluorescence can be reduced under the UV lamp upon increasing the concentration of the target (insets of Figure 5A). Correspondingly, the yellow color of DAP becomes lighter due to the stronger inhibition of Pd/Pt NFs catalyst by Ag^+^ (insets of Figure 5C). Therefore, the concentration of silver ions can be clearly judged based on the color and fluorescence.

### 3.6. Ag^+^ Detection in Real Samples

In daily life, Ag^+^ pollution can cause poisoning and seriously affect the ecological environment and human health. Therefore, the detection of Ag^+^ in solution via a simple and fast method is of great significance to human health and ecological environment. In order to verify the applicability of this method in practical application, three kinds of commercially available drinking water were selected as the real samples. Then, different concentrations of Ag^+^ were added into these samples for recovery experiments. As demonstrated in Table 2 and Table 3, the recovery rates of the three kinds of drinking water range from 91.2% to 118.0%, from 88.1% to 110.0% and from 94.6% to 114.0%, respectively. Moreover, the relative standard deviation (RSD) is determined within the range from 1.21% to 5.61%, which shows that this method has good feasibility. This test verifies the applicability and accuracy of the method. The results of the fluorescence and colorimetric dual-mode assay prove that the developed method is in good agreement. All of the above results definitely show that this detection method can be used as a reliable application of Ag^+^ in a practical sample testing strategy.

## 4. Discussion

In summary, a simple yet sensitive dual-mode fluorescence and colorimetric Ag^+^ detection strategy based on the sensing system of OPD and Pd/Pt NFs was realized in this study. Pd/Pt NFs with peroxidase-like activity can effectively oxidize OPD into DAP, which has a high fluorescence emission and UV-vis absorbance. In fluorescence analysis, when Ag^+^ are present in the sample, Pd/Pt NFs can bind specifically to Ag^+^, thus inhibiting the catalytic activity of Pd/Pt NFs. The concentration of Ag^+^ is determined via the monitoring of the fluorescence produced by DAP. Meanwhile, the concentration of Ag^+^ can also be determined based on the UV-vis absorption of DAP. Both the fluorescence intensity and UV-vis absorbance of the system can be linearly reduced for the quantitative detection of Ag^+^, and the LOD of the fluorescence and colorimetric channels are 5.8 nM and 46.9 nM, respectively. Fluorescence analysis has higher sensitivity and selectivity compared to the UV spectrum, while the UV-vis absorption can provide more accurate quantitative analysis results. The combination of two different detection methods improves the reliability of the analysis results, avoiding false negative and false positive results and expanding the application range. Eventually, the feasibility of this method is verified by detecting Ag^+^ in real samples, with satisfactory results. The constructed fluorescence and UV-vis dual-channel sensor has the benefits of high sensitivity, simple operation, short time, low cost and visualized detection, thus providing a new method for the rapid detection of Ag^+^ in food. 

## Figures and Tables

**Table 1:** Comparison of our proposed detection method with other representative strategies.

Method	LOD (nM)	Linear Range (μM)	Ref.
g-C_3_N_4_	6.31	0–100	[27]
TTP	76.5	0–2	[28]
TA/PUL-AuNPs	30.8	0.1–150	[29]
Coumarinyl ligand	8.8	2–20	[30]
PEI-ARD-60	90.7	0.03–1	[31]
OPD-Pd/Pt NFs	5.8	0–0.25	This work

**Table 2:** Ag^+^ detection in real samples (fluorescent method).

Sample	Add (μM)	Found (μM)	Recovery (%)	RSD (%, *n* = 3)
Water A	0	-	-	-
	0.1	0.0912	91.2	2.02
	0.2	0.214	107.0	1.21
Water B	0	-	-	-
	0.1	0.0881	88.1	3.63
	0.2	0.205	102.5	2.97
Water C	0	-	-	-
	0.1	0.0975	97.5	2.38
	0.2	0.197	98.5	2.68

**Table 3:** Ag^+^ detection in real samples (colorimetric method).

Sample	Add (μM)	Found (μM)	Recovery (%)	RSD (%, *n* = 3)
Water A	0	-	-	-
	0.1	0.118	118.0	4.84
	0.2	0.219	109.5	1.99
Water B	0	-	-	-
	0.1	0.110	110.0	5.19
	0.2	0.213	106.5	2.10
Water C	0	-	-	-
	0.1	0.0946	94.6	5.61
	0.2	0.228	114.0	4.41

## Data Availability

Data are contained within the article.
